# Hydrogels Based on Alginates and Carboxymethyl Cellulose with Modulated Drug Release—An Experimental and Theoretical Study

**DOI:** 10.3390/polym13244461

**Published:** 2021-12-20

**Authors:** Cătălina Anișoara Peptu, Elena Simona Băcăiță, Corina-Lenuta Savin (Logigan), Marian Luțcanu, Maricel Agop

**Affiliations:** 1 Department of Natural and Synthetic Polymers, Faculty of Chemical Engineering and Environmental Protection, “Gheorghe Asachi” Technical University of Iasi, 71, Prof. Dr. Docent DimitrieMangeron Street, 700050 Iasi, Romania; catipeptu@ch.tuiasi.ro (C.A.P.); savincorina@yahoo.com (C.-L.S.); 2 Department of Physics, Faculty of Machine Manufacturing and Industrial Management, “Gheorghe Asachi” Technical University of Iasi, Bd. Prof. Dr. Docent Dimitrie Mangeron73, 700050 Iasi, Romania; maricel.agop@academic.tuiasi.ro; 3 Materials Science Department, Faculty of Materials Science and Engineering, “Gheorghe Asachi” Technical University of Iasi, 71, Prof. Dr. Docent Dimitrie Mangeron Street, 700050 Iasi, Romania; marian.lutcanu@staff.tuiasi.ro

**Keywords:** alginic acid, carboxymethyl cellulose, films, epichlorohydrin, drug delivery, fractalization

## Abstract

New hydrogels films crosslinked with epichlorohydrin were prepared based on alginates and carboxymethyl cellulose with properties that recommend them as potential drug delivery systems (e.g., biocompatibility, low toxicity, non-immunogenicity, hemostatic activity and the ability to absorb large amounts of water). The characterization of their structural, morphological, swelling capacity, loading/release and drug efficiency traits proved that these new hydrogels are promising materials for controlled drug delivery systems. Further, a new theoretical model, in the framework of Scale Relativity Theory, was built with to offer insights on the release process at the microscopic level and to simplify the analysis of the release process.

## 1. Introduction

In recent years, significant progress has been made in the field of biology, medicine and materials science, which has led to the development of innovative systems, including films, hydrogels and micro- and nanoparticulate systems based on natural/synthetic polymers as drug carriers. The advantages of such drug delivery systems are the improved therapeutic efficacy of drugs and reduced side effects and associated costs [1,2,3]. Generally, polymeric films are biocompatible and can be used as supports for the controlled release of bioactive compounds. They can be obtained through the chemical and/or physical crosslinking of hydrophilic polymers, and their physical-chemical properties depend on the type and density of crosslinking in addition to the molecular weight and chemical composition [4].

In recent years, a wide variety of sources for drug delivery systems have been developed and applied, such as proteins, polysaccharides, synthetic polymers and organo–inorganic hybrids. Of these, polysaccharides present important advantages as they can be obtained from various natural renewable resources (ocean and plant) and they present an abundance of functional groups, such as hydroxyl (-OH), amino (-NH2) and carboxyl (-COOH), that make them suitable for chemical modification in order to adjust certain properties, biocompatibility, bioactivity and nontoxicity [5].

Alginates (AG) are natural hydrophilic colloidal polysaccharides obtained from various species of brown algae. They are extensively investigated and used for many biomedical applications, due to several unique properties, including biocompatibility, low toxicity, non-immunogenicity, hemostatic activity and accessibility at low prices. In addition to these, they form gels easily by adding bivalent cations as Ca^2+^. Alginates are known as natural block copolymers containing 1,4-linked residues of β-D-manuronate (M) and α-L-guluronate (G). The content in the residues of D-manuronate and α-L-guluronate alginates and the length of each block may differ depending on the species of algae used for their extraction. Alginates are excellent candidates for the development of different systems, such as films, membranes, hydrogels, particles and nanofibers [1,4,6].

Carboxymethyl cellulose (CMC) is another polysaccharide widely used in the biomedical field. It is cellulosic, which, when in contact with aqueous media, leads to the formation of colloidal solutions that contain, at low concentrations, filiform macromolecules and gels at high concentrations. CMC solutions are viscous at acidic pH, and the viscosity increases with the decreasing pH value. The solution viscosity decreases with temperature but returns to the initial value after cooling. CMC has desirable characteristics for a drug delivery system, such as biocompatibility, non-toxicity and the ability to absorb large amounts of water, and CMC has been applied as a stabilizer and as protective colloids in pharmaceuticals [7,8,9].

Despite the excellent properties of both alginate and carboxymethyl cellulose, their combination as hydrophilic/hydrophilic polymers in the formation of new materials represents a challenging task due to their high-water solubility [10]. To overcome this limitation, an alternative is to add in the polymeric matrix a crosslinking agent, such as epichlorohydrin (ECH). As result, a film with increased resistance in contact with aqueous media, and thus a higher tensile strength, is obtained. Moreover, the crosslinking step allows control of the release rate of bioactive compounds through the film matrix [11] leading to new materials with synergetic properties.

Aiming to exploit the AG and CMC properties that recommend them as potential drug delivery systems (i.e., biocompatibility, low toxicity (AG and CMC), non-immunogenicity, hemostatic activity, accessibility at low prices (AG) and the ability to absorb large amounts of water (CMC)), new hydrogel films, based on AG and CMC with epichlorohydrin (ECH) as a crosslinker, were prepared and characterized in order to evaluate their properties as drug delivery systems [12].

Fourier-transform infrared spectroscopy (FT-IR) and scanning electron microscopy (SEM) were used to characterize the structures and morphology. Further, the swelling capacity and loading/release drug efficiency, using metronidazole (MT) as a model drug, were evaluated. The further goal of the present study is to use these formulations as wound dressing materials and, for this purpose in this preliminary study, MT was chosen since it is widely used for its ability to treat a large variety of infections.

## 2. Experimental Procedure

### 2.1. Materials

The following materials: AG (alginic acid sodium salt from brown algae with medium viscosity, Brookfield viscosity 2% in water at 25 °C, >2000 cps), CMC (medium viscosity, sodium (Na) 6.5–9.5%. Brookfield viscosity 2% in water at 25 °C, 400–800 cps. Degree of substitution 0.65–0.90), NaOH and ECH were purchased from Sigma-Aldrich; Metronidazole 99% was purchased from Alfa Aesar; and Milli-Q ultrapure distilled water (DW) was purchased from Merck Chemicals (Darmstadt, Germany). Epichlorohydrin (ECH) was used as a crosslinking agent without any pretreatment. All chemical reagents were of analytical-grade purity and were used without further purification.

### 2.2. Instrumentation

The film morphology was investigated via the SEM technique: films were fixed on an aluminum stub and coated with a 7 nm thick gold layer using a Cressington 108 device before observation, and SEM images were recorded with a HITACHI SU 1510 (Hitachi SU-1510, Hitachi Company, Tokyo, Japan) Scanning Electron Microscope at an accelerating voltage of 25 kV.

Fourier Transform Infrared Spectroscopy spectra of films were recorded with a DIGILAB SCIMITAR FTS 2000 spectrometer. The samples were prepared as KBr pellets and scanned over the wave number range of 4000–450 cm^−1^ at a resolution of 4.0 cm^−1^.

### 2.3. Methods

#### 2.3.1. Preparation of AG/CMC Films

The AG/CMC films were synthesized via the Jeong et al. method with certain modifications. Films based on AG/CMC were prepared as follows: 500 mg of CMC was dissolved in 10, 15 and 20 mL of 9 wt.% NaOH solution. Subsequently, 500 mg of AG was added to the CMC solution, and, after the complete dissolution of both polymers, 1.5 mL of ECH was slowly added to the solution while stirring at 600 rpm at 25 °C for 25 min. The resulting films were washed with ethanol solution. Finally, all the samples were freeze-dried. The initial reactants composition is shown in Table 1. The percent hydrogel yield was obtained using the following equation:

(1) $$Films\;yield,\%=\frac{Dry\;weight\;of\;film}{Total\;weight\;of\;\mathrm{reactant}\;\mathrm{in}\;\mathrm{feed}}\times 100.$$

#### 2.3.2. Evaluation of Swelling Characteristic

The film swelling properties were evaluated in order to predict their loading and releasing capacity. All synthesized films were immersed in vials (glass bottles of 10 mL) containing 3 mL of DW. They were maintained at room temperature for 4 days until the swelling equilibrium was reached. The swelling properties were monitored at different times, the films were weighed, and the DW was gently removed from the vial and the film surface with laboratory tissues.

The water swelling ratio was determined with the equation:

(2) $$Q(\%)=\frac{w_{s}-w_{0}}{w_{0}}\times 100$$

where $w_{s}$ is the weight of the swollen probe and $w_{0}$ is the weight of the dry probe.

#### 2.3.3. Evaluation of Metronidazole Loading and Release

Drug loading and release studies were performed via a diffusional mechanism [13]. MT as a drug model was used. First, samples of dried films were placed in a glass vial (10 mL volume), and 3 mL of MT aqueous solution (10 mg/mL) was added and maintained at room temperature. The glass vials containing the films and MT solution were left in the solution during 48 h and gently stirred by a small Teflon bar. The quantity of MT retained was determined spectrophotometrically based on the calibration curve previously obtained [14]. After 2 days, the MT-loaded films were washed with DW, and the remaining water on the films surface was gently wiped away with a laboratory tissue.

Next, the release profiles of MT were evaluated in DW at room temperature. Periodically, 20 µL of volume-releasing solution was withdrawn from the DW. The volume of DW was held constant by adding fresh DW. The amount of released MT was calculated spectrophotometrically with a spectrophotometer, UV–VIS NanoDrop ND −1000, at a wavelength of 320 nm. All measurements were performed in triplicate and averaged. The equations used are as follows, where *W_MT_* is the amount of *MT*:

(3) $$Drug\;loading\;\mathrm{efficiency}\;(\%)=\frac{W_{MTcrude}-W_{MT\mathrm{supernatant}}}{W_{MTplain}}\times 100$$

(4) $$\mathrm{Release}\;\mathrm{efficiency}\;(\%)=\frac{W_{MTreleased}}{W_{MTtotal}}\times 100\;$$

## 3. Results and Discussions

A first important step in the design of a hydrogel type system is represented by the selection of precursors and the method of preparation, which strongly influences the speed and mechanism of the release of bioactive compounds encapsulated in the hydrogel. In order to analyze the influence of reaction parameters on the final hydrogel properties and to select the best formulation for drug delivery systems, varied ratios between AG and CMC were tested regarding the ECH concentration and NaOH reaction volume. The sample F1, which was prepared using the following parameters, AG/CMC ratio 1:1, 0.02 mol of ECH, 15 mL of NaOH 9 wt.% and a 25 min reaction time, was chosen for further experiments due to the medium values of most of the considered variable parameters (Table 1).

After the purification step with ethanol and freeze drying, the recovered fractions were found to be relatively high at around 90% in the case of sample F2, with a ratio of 1:2 (AG: CMC), and lower than 43% for the F7 sample, which had a high quantity of ECH crosslinking agent. The high recovered fraction in the case of sample F2 may be explained by the high CMC quantity compared to AG, which could provide a high mechanical strength of the film, due to a longer polymer chain. In the case of sample F2, the amount of crosslinker, referring to the total reaction component, was reduced, which favors the obtaining of a higher yield compared to the F7 sample that had a high ECH concentration. Therefore, we can state that the yields of films are dependent on the polymer and crosslinking agent concentration (see Table 1).

### 3.1. Structural Characterization

#### 3.1.1. FTIR Characterization

**Figure 1:**
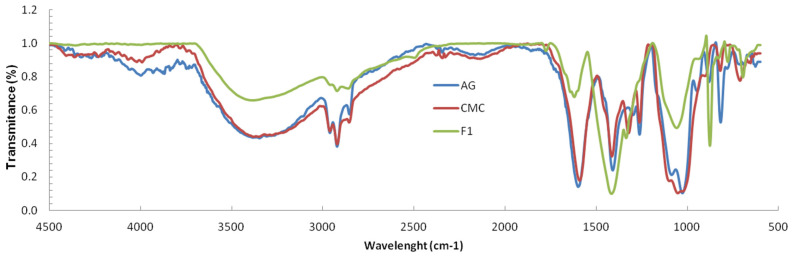
Fourier transform infrared (FTIR) spectra of the AG, CMC and F1 synthesized film.

The Fourier transform infrared (FTIR) spectra of the AG, CMC and synthesized film were recorded and compared (Figure 1). First, the recorded AG spectra revealed important absorption bands concerning the hydroxyl and carboxylic functional groups. In the range of 3107–3579 cm^−1^, stretching vibrations of O–H bonds of AG appeared. Stretching vibrations of C–H were observed at 2854–2960 cm^−1^. The bands observed at 1598 and 1409 cm^−1^ were attributed to asymmetric and symmetric stretching vibrations of COO– groups. The bands at 1028 and 947 cm^−1^ were attributed to the C–O stretching with contributions from C–C–H and C–O–H deformation.

For CMC, a characteristic peak at 2922 cm^−1^ was observed corresponding to C–H anti-symmetrical stretching. The bands observed at 1413 and 1591 cm^−1^ were attributed to the carboxylate group stretching vibrations (symmetric and asymmetric). Moreover, a visible band was observed at 3350 cm^−1^ characteristic to –OH groups.

In the case of the synthesized film spectrum, significant differences in bands were observed, compared with those of the plain AG and CMC, as characteristic peaks were observed at 1620, 1336 and 1058 cm^−1^, which can be attributed to the stretching modes of the (C=O), (C-C-O) and (O-C-C) bonds of the ester group, respectively. At 877 cm^−1^, a band specific to the C-H bending was observed. Further, at 1415 cm^−1^, a higher intensity of the O-H bending peak compared to AG and CMC spectrum, which may be attributed to a cross-linked network, was observed.

#### 3.1.2. Morphological Characterization by SEM

**Figure 2:**
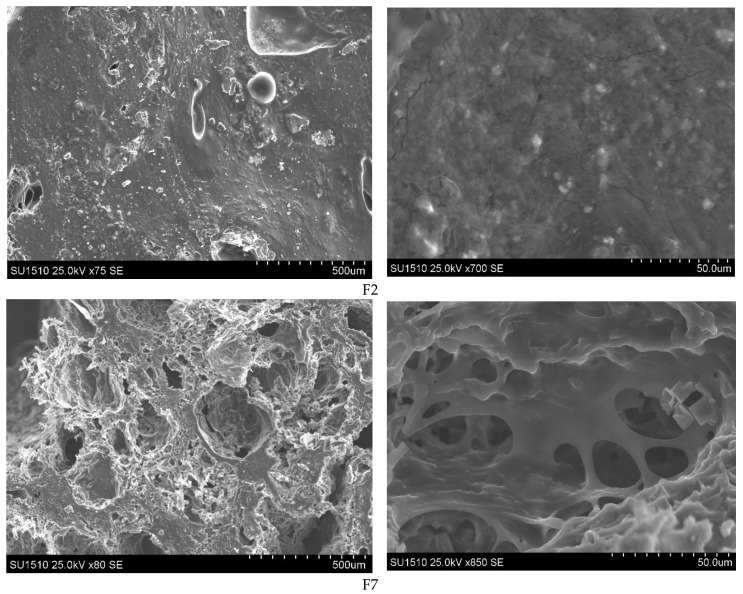
SEM images of the AG/CMC films (samples F2 and F7).

The SEM technique was used to visualize the morphological characteristics of lyophilized hydrogel films. Images of the films surface and section morphology are highlighted in Figure 2, for F2 and F7 samples, and in Figure S1, for all other samples. We observed that all films were compact and presented surfaces with aggregates and roughness. However, as can be observed from the cross-section images, all films presented areas with large pores of irregular sizes and shapes. 

Comparing SEM pictures for the F2 and F3 samples, one can see that the porosity of the film decreased with increasing polymer concentration; therefore, we can state that the formation of the porous structures depends on the polymer’s initial concentration. Moreover, SEM images revealed that the F1 sample had a surface without pores in contrast with samples F6 and F7, which had porous structures at the same polymer concentration. In conclusion, the morphology of polymer hydrogels, which influences further properties, can be adjusted by varying the crosslinking reaction parameters.

### 3.2. Evaluation of Hydrogels Behavior in Aqueous Media

Considering the hydrophilicity of the initial polymers, swelling properties of the AG/CMC films are a key factor regarding their ability to load/release bioactive components. Swelling studies were performed for all prepared samples in DW. The swelling ratios Q(%) were calculated, and the results can be seen in Table 2.

The obtained results reveal a connection between the swelling degree and sample preparation parameters, such as the polymer and crosslinker concentration and reaction volume. The best swelling ratio of 1273%, indicating high superabsorbent properties, was observed for the F6 sample (1:1 (AG: CMC) molar ratio and 6.6% polymer concentration). This result was expected, since the SEM images revealed, for the F6 sample, a very porous structure. Still, despite its porous structure, as shown by SEM images, the sample F7 had a swelling ratio of only 362%.

**Figure 3:**
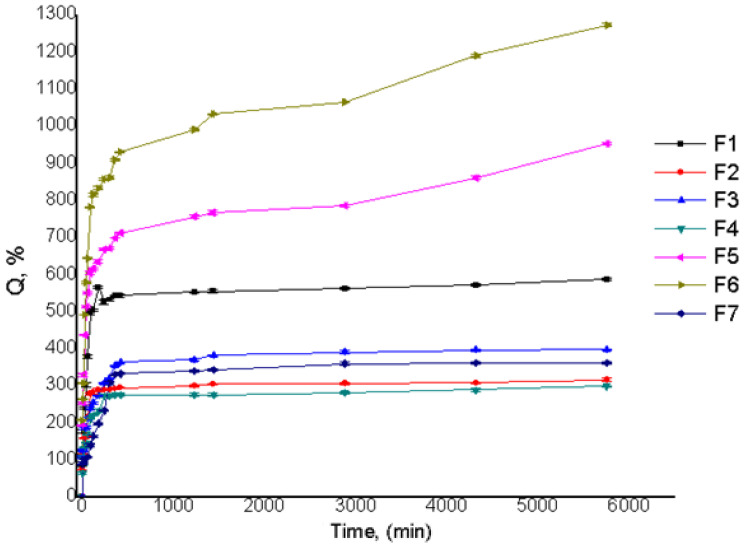
Film swelling ratios for films with different reaction parameters.

This can be explained due to the higher ECH crosslinker concentration of sample F7, which determined a more extensively crosslinked polymeric network. The polymer concentration influenced the swelling ratio: at low polymer concentrations, higher swelling ratios were obtained compared to the samples with high concentrations that showed low swelling ratios. These results demonstrated that the swelling ratio Q(%) was dependent both on the polymer and crosslinking agent concentration (Figure 3).

### 3.3. Evaluation of Metronidazole Loading and Release Kinetics

The potential of AG/CMC hydrogels as drug delivery systems was investigated by evaluating their drug loading and release efficiency. MT, a limited spectrum antibiotic that actively deters the growth of protozoa, anaerobic gram-positive and anaerobic gram-negative bacteria, was used as the model drug. All prepared films were loaded with a MT solution (10 mg/mL), and subsequently the released MT from the loaded films in DW was determined.

**Figure 4:**
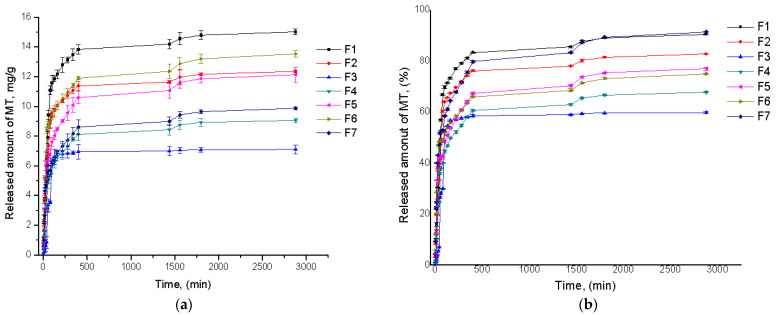
The MT release kinetics from synthesized films expressed in terms of mg/g (**a**) and released efficiency (**b**).

The amount of MT loaded in hydrogel films after 48 h was between 10.8 mg and 18 mg, corresponding to a loading efficiency between 40% and 64% (Table 3). As expected, sample F6, which had the higher swelling ratio, also presented the higher MT-loading capacity (18 mg, 64%) in comparison to sample F7 (10.8 mg, 40%) most likely due to a stronger complexation capability of sample F6 compared to F7. We can conclude that the amount of MT loaded in hydrogel films is dependent on the polymers and ECH concentration used in the preparation procedure (Figure 4).

Regarding the drug release capacity, all hydrogel films released between 9.88 mg/g and 15.03 mg/g MT or, in terms of efficiency, between 59% and 91% MT (Table 3), demonstrating a good ability to release the active compound. The release kinetics showed an initial burst release phase in the initial approximately 100 min, followed by a slower release phase within the next 9 h, characterized by a final constant release until 48 h.

By analyzing the influence of synthesis parameters on the film’s release capacity, we noticed that the sample that had the highest amount of released MT was F1 (15.03 mg/g), in contrast to sample F6 (13.54 mg/g). This result, somehow unexpected, may be due to the host–guest complex formation between hydrogel network and MT: a high amount of MT inside the film may have caused a recomplexation between the crosslinked hydrogel film and MT. These results suggest that samples F1 and F6 are more effective as drug delivery systems that are capable of encapsulating a large amount of MT and releasing it effectively.

## 4. Theoretical Evaluation of Drug Release

### 4.1. Justification for the Need for a New Mathematical Model

The experimental results, previously discussed, led to some unexpected results in respect to the dependence of drug release on the crosslinker amount. For example, the expected behavior was that, as the amount of crosslinker increased, the swelling ratio and the amount of drug loaded/released into/from the polymer network would decrease due to the higher network strength. The experimental results confirmed this hypothesis for the swelling ratio and the loaded drug but failed for the released drug amount/efficiency; moreover, the expected similarity in terms of swelling and loading/releasing efficiency was not encountered (Table 4).

The given explanation implies a certain degree of uncertainty, i.e., a possible host–guest complex formation between the hydrogel network and drug. The above issue raises, once again, the problem of the complexity of the release processes and the interdependence of the variables that appear: the strength of the hydrogel network and the concentration of the loaded drug; in turn, the strength of the network is determined by the amount of crosslinker used, the polymer concentration, their molar ratio etc.

### 4.2. The Theoretical Model of Drug Release in the Framework of Scale Relativity Theory

As a consequence of the information presented above, the need to understand the release process adds the idea of building a theoretical model of drug release able to offer insights on the release process at microscopic level and to simplify the analysis of the release process. The main purpose of the presented model is to be an alternative to the classical models of drug release, which analyze the phenomenon only empirically at the macroscopic level [15] as if ignoring that matter obtains its properties from the molecules it is made of and their interactions.

The starting point of the theoretical model is the Scale Relativity Theory [16,17] that, in recent years, has been used successfully in describing the dynamics of complex systems, such as nanofluids [17] and plasma [18,19,20,21,22,23]. The hypothesis underlying this theory is that the entities of any complex system move on continuous and non-differential curves named fractal curves, i.e., three dimensional fractured lines, whose non-linearity is dependent and proportional with the number of interactions within the system. In this context, the fractalization degree will be defined as a measure of the system complexity, and the physical quantities, characterizing the system evolution, will be fractal functions dependent both on the spatio-temporal coordinates and resolution scales [24].

Further, we will use this hypothesis in the case of our system, which will be considered as a complex system consisting of a hydrogel, drug and release medium. Previous studies showed that the theoretical models built on this hypothesis allowed insights concerning drug release at the microscopic level, which has been unaddressed thus far due to the complexity of the phenomena involved. For example, this revealed that, in the case of polymeric particles based on chitosan and gelatin, the trajectories of drug molecules follow cnoidal oscillation modes [25], while, for polysaccharide-based hydrogels, their trajectories evolved from a normal period doubling state towards damped oscillating via strong modulated dynamics [26]. 

For chitosan-gelatin and chitosan-poly(vinyl alcohol) hydrogels, analyzing the release kinetics at long time scales (i.e., up to 25 days), when the hydrogel films degrade, it was found that the release efficiency was dependent on the time and system nonlinearity, i.e., the fractalization degree [27]. Moreover, it was found that, along with the evolution through all the phases of the release process (i.e., burst, swelling, equilibrium and degradation phases), the fractalization degree increased, thus, reflecting the increasing system complexity [28].

In this study, we will consolidate this hypothesis by analyzing the release dynamics in two directions: (i) the release dynamics in the Schrodinger fractal representation and (ii) the release dynamics in the Mandelung fractal hydrodynamic representation [24].

In our opinion, the two representations describing the dynamics of release are not excluded; on the contrary, they are complementary. As the problem of compatibility of the two representations in the description of the release dynamics has not been analyzed thus far, next we will analyze such a problem and its implications.

#### 4.2.1. Two Models of Describing the Release Dynamics through the Scale Relativity Theory

In all the above assumptions, the motion equation of any of the system component (either drug and either release medium molecules) can be written in the form:

(5) $$\frac{d\overset{\wedge }{V^{i}}}{dt}=\partial_{t}\overset{\wedge }{V^{i}}+\overset{\wedge }{V^{l}}\partial_{l}\overset{\wedge }{V^{i}}+\frac{1}{4}{\left(dt\right)}^{{}^{\left[\frac{2}{f\left(\alpha \right)}\right]-1}}D^{lk}\partial_{l}\partial_{k}\overset{\wedge }{V^{i}}=0$$

where

(6) $$\begin{array}{c} \overset{\wedge }{V^{l}}=V_{D}^{l}-V_{F}^{l}\\ D^{lk}=d^{lk}-i\overset{\wedge }{d^{lk}}\\ d^{lk}=\lambda_{+}^{l}\lambda_{+}^{k}-\lambda_{-}^{l}\lambda_{-}^{k}\\ \overset{\wedge }{d^{lk}}=\lambda_{+}^{l}\lambda_{+}^{k}+\lambda_{-}^{l}\lambda_{-}^{k}\\ \partial_{t}=\frac{\partial }{\partial t},\partial_{l}=\frac{\partial }{\partial x^{l}},\partial_{l}\partial_{k}=\frac{\partial }{\partial x^{l}}\frac{\partial }{\partial x^{k}},\;i=\sqrt{-1},\;l,k=1,2,3 \end{array}$$

where $x^{l}$ is the multifractal spatial coordinate, t is the non-fractal time with the role of an affine parameter of the motion curves, $\overset{\wedge }{V^{l}}$ is the complex velocity, $V_{D}^{l}$ is the differential velocity independent of scale resolution, $V_{F}^{l}$ is the non-differential/fractal velocity dependent of scale resolution, dt is the scale resolution, f(α) is the singularity spectrum of order α, α is the singularity index and a function of fractal dimension $D_{f}$, $D^{lk}$ is the constant tensor associated with the differentiable–non-differentiable transition, $\lambda_{+}^{l}\left(\lambda_{+}^{k}\right)$ is the constant vector associated with the backward differentiable-non-differentiable dynamic processes, $\lambda_{-}^{l}\left(\lambda_{-}^{k}\right)$ is the constant vector associated with the backward differentiable-non-differentiable dynamic processes, and F is a multifractal function.

In the case of our drug release system, multifractalization will be considered by means of Markovian stochastic processes, i.e., processes for which the probability of possible “events” in system evolution depends only on the state attained in the previous “event”. From a mathematical point of view, the following restrictions must be fulfilled:

(7) $$\lambda_{+}^{i}\lambda_{+}^{l}=\lambda_{-}^{i}\lambda_{-}^{l}=2\lambda \delta^{il},\;\;i,l=1,2,3$$

where λ is a constant associated with the differentiable–non-differentiable transition, which, in the case of release dynamics, is actually the transition from Fickian to non-Fickian diffusion, and $\delta^{ir}$ is Kronecker’s pseudo-tensor [16,17,24].

Imposing the restrictions from (7), Equation (5) becomes:

(8) $$\frac{d\overset{\wedge }{V^{i}}}{dt}=\partial_{t}\overset{\wedge }{V^{i}}+\overset{\wedge }{V^{l}}\partial_{l}\overset{\wedge }{V^{i}}-i\lambda {\left(dt\right)}^{\left[\frac{2}{f\left(\alpha \right)}\right]-1}\partial_{l}\partial^{l}\overset{\wedge }{V^{i}}=0$$

where $\partial_{t}\overset{\wedge }{V^{i}}$ represents the local multifractal acceleration, $\overset{\wedge }{V^{l}}\partial_{l}\overset{\wedge }{V^{i}}$ the multifractal convection and $i\lambda {\left(dt\right)}^{\left[\frac{2}{f\left(\alpha \right)}\right]-1}\partial_{l}\partial^{l}\overset{\wedge }{V^{i}}$ the multifractal dissipation. Equality with zero from Equation (8) reflects the fact that the local multifractal acceleration, the local multifractal convection and the multifractal dissipation are in equilibrium at all points of the motion curve. Moreover, the existence of the complex dissipation term confirms that the multifractal fluid with which the drug release complex system can be assimilated exhibits memory according to the assumption of Markovian stochastic processes.

Considering that, in the release flow, each element of the moving fluid undergoes no net rotation (i.e., rotational movements of system structural units), the complex velocity field from (6) becomes

(9) $$\overset{\wedge }{V^{i}}=-2i\lambda {\left(dt\right)}^{\left[\frac{2}{f\left(\alpha \right)}\right]-1}\partial^{i}\ln\Psi$$

where

(10) $$\chi =-2i\lambda {\left(dt\right)}^{\left[\frac{2}{f\left(\alpha \right)}\right]-1}\ln\Psi$$

is the complex scalar potential of the complex velocity fields from (6), and Ψ is the state function that describes the equilibrium state of the system. Substituting (9) in (8) and using the mathematical procedure from [24], Equation (8) takes the form of the multifractal Schrödinger type equation:

(11) $$\lambda^{2}{\left(dt\right)}^{\left[\frac{4}{f\left(\alpha \right)}\right]-2}\partial^{l}\partial_{l}\Psi +i\lambda {\left(dt\right)}^{\left[\frac{2}{f\left(\alpha \right)}\right]-1}\partial_{t}\Psi =0$$

Therefore, for the complex velocity field (9), the dynamics of any complex system entities are described through multifractal Schrödinger “regimes”, i.e., Schrödinger equations at various scale resolutions.

From such a perspective, the meaning of Ψ can be given based on a conservation law. Indeed, multiplying Equation (11) with $\bar{\Psi }$ (complex conjugate of Ψ) and the complex conjugate of Equation (11) with Ψ, followed by their difference, the multifractal law of conservation for the state density is obtained in the form [29]:

(12) $$\partial_{t}\rho +\partial_{l}J^{l}=0$$

where

(13) $$\rho =\Psi \bar{\Psi ,}J^{l}=\frac{\lambda {\left(dt\right)}^{\left[\frac{2}{f\left(\alpha \right)}\right]-1}}{i}\left(\bar{\Psi }\partial^{l}\Psi -\Psi \partial^{l}\bar{\Psi }\right)$$

In the relations (12) and (13), ρ is the multifractal state density, while $J^{l}$ is the multifractal current density. In the case of the existence of a constraint, such as the hydrogel network-drug interactions, in the form B=rotA, the usual derivative from the Scale Relativity Theory is substituted with the covariant derivative, $\nabla^{l}$:

(14) $$\nabla^{l}=\partial^{l}-i\frac{g}{2}A^{l}$$

where $A^{l}$ is a vector potential and g is a coupling constant between Ψ and $A^{l}$. In such a context, (12) maintains its validity, with the difference that $J^{l}$ from (13) can be expressed as

(15) $$J^{l}=\frac{\lambda {\left(dt\right)}^{\left[\frac{2}{f\left(\alpha \right)}\right]-1}}{i}\left(\bar{\Psi }\partial^{l}\Psi -\Psi \partial^{l}\bar{\Psi }\right)-gA^{l}\Psi \bar{\Psi }$$

Moreover, if Ψ is chosen of the form (Mandelung’s choice):

(16) $$\Psi =\sqrt{\rho }e^{is}$$

where $\sqrt{\rho }$ is the amplitude of the state quantities and s the phase, then the complex velocity field (9) takes the explicit form:

(17) $$\overset{\wedge }{V^{i}}=2\lambda {\left(dt\right)}^{{}^{\left[\frac{2}{g\left(\alpha \right)}\right]-1}}\partial^{i}s-i\lambda {\left(dt\right)}^{{}^{\left[\frac{2}{g\left(\alpha \right)}\right]-1}}\partial^{i}\ln\rho ,$$

which leads to the determination of velocity fields:

(18) $$\begin{array}{l} V_{D}^{i}=2\lambda {\left(dt\right)}^{\left[\frac{2}{f\left(\alpha \right)}\right]-1}\partial^{i}s\\ V_{F}^{i}=\lambda {\left(dt\right)}^{\left[\frac{2}{f\left(\alpha \right)}\right]-1}\partial^{i}\ln\rho \end{array}$$

By (17) and (18) and using the mathematical approaches from [24,29], the Equation (8) reduces to the multifractal Mandelung’s equations:

(19) $$\partial_{t}V_{D}^{i}+V_{D}^{l}\partial_{l}V_{D}^{i}=-\partial^{i}Q$$

(20) $$\partial_{t}\rho +\partial_{l}\left(\rho V_{D}^{l}\right)=0$$

where Q denotes the multifractal specific potential:

(21) $$Q=-2\lambda^{2}{\left(dt\right)}^{\left[\frac{4}{f\left(\alpha \right)}\right]-2}\frac{\partial^{l}\partial_{l}\sqrt{\rho }}{\sqrt{\rho }}=-V_{F}^{i}V_{F}^{i}-\frac{1}{2}{\left(dt\right)}^{\left[\frac{2}{f\left(\alpha \right)}\right]-1}\partial_{l}V_{F}^{l}$$

Equation (19) corresponds to the multifractal specific momentum conservation law, while Equation (20) corresponds to the multifractal density conservation law [24]. The specific multifractal potential Q expressed by (21) implies the existence of a specific multifractal force:

(22) $$F^{i}=-\partial^{i}Q=-2\lambda^{2}{\left(dt\right)}^{\left[\frac{4}{f\left(\alpha \right)}\right]-2}\partial^{i}\frac{\partial^{l}\partial_{l}\sqrt{\rho }}{\sqrt{\rho }}$$

that quantifies the multifractality degrees of the motion trajectories.

Therefore, for the complex velocity fields (18), the dynamics of any complex system are described through multifractal Madelung “regimes” (i.e., Madelung equations at various scale resolutions). In this last context, the following consequences result:

(i) Any complex system entities are in permanent interaction with a multifractal medium through the multifractal specific force (18) [29].
(ii) Any complex system can be identified with a multifractal fluid, the dynamics of which is described by the multifractal Madelung model (Equations (19) and (20)).
(iii) The velocity field $V_{F}^{i}$ does not represent the contemporary dynamics; since $V_{F}^{i}$ is missing from (20), this velocity field contributes to the transfer of the multifractal specific momentum and to the multifractal energy.
(iv) Any analysis of Q should consider the “self” nature of the multifractal specific momentum transfer; then, the conservation of the multifractal energy and the multifractal momentum ensure the reversibility and the existence of the multifractal eigenstates.
(v) If a multifractal tensor is considered:
  (23) $$\overset{\wedge }{\tau^{il}}=2\lambda^{2}{\left(dt\right)}^{\left[\frac{4}{f\left(\alpha \right)}\right]-2}\rho \partial^{i}\partial^{l}\ln\rho$$
  the equation defining the multifractal “forces” that derive from Q can be written in the form of a multifractal equilibrium equation:
  (24) $$\rho \partial^{i}Q=\partial_{l}\overset{\wedge }{\tau^{il}}.$$

Since $\overset{\wedge }{\tau^{il}}$ can be also written in the form:

(25) $$\overset{\wedge }{\tau^{il}}=\eta (\partial_{l}V_{F}^{i}+\partial_{i}V_{F}^{l})$$

with

(26) $$\eta =\lambda {\left(dt\right)}^{\left[\frac{2}{f\left(\alpha \right)}\right]-1}\rho$$

a multifractal linear constitutive equation for a multifractal “viscous fluid” can be highlighted. In such a context, the coefficient η can be interpreted as multifractal dynamic viscosity coefficient of multifractal fluid.

#### 4.2.2. The Compatibility of the Two Models of Describing the Release Dynamics through the Scale Relativity Theory

As anticipated, the two multifractal descriptions of the dynamics of complex systems, the multifractal Schrödinger description and the multifractal Madelung one, are not mutually exclusive but, on the contrary, are complementary. Let us explain this on the basis of the following hypotheses:

(a) The dynamics of any complex system, independent of the two scale resolutions (differentiable and non-differentiable scale resolutions), are one-dimensional dynamics. It thus results that the conservation law for the multifractal states density (12) becomes
  (27) $$\partial_{t}\rho +\partial_{x}\left(J_{x}\right)=0$$
  where
  (28) $$J_{x}=\frac{\lambda {\left(dt\right)}^{\left[\frac{2}{f\left(\alpha \right)}\right]-1}}{i}\left(\bar{\Psi }\frac{d\Psi }{dx}-\Psi \frac{d\bar{\Psi }}{dx}\right)-gA_{x}\Psi \bar{\Psi }$$
  In particular, for the state function in the form (16), Equation (28) becomes
  (29) $$J_{x}=2\lambda {\left(dt\right)}^{\left[\frac{2}{f\left(\alpha \right)}\right]-1}\left(\frac{ds}{dx}\right)\rho -gA_{x}\rho$$
(b) The synchronization of the dynamics of any complex system at the two scale resolutions is achieved by “compensating” the speed fields $V_{D}^{l}$ and $V_{F}^{l}$ given by the restriction:
  (30) $$V_{D}^{l}=-V_{F}^{l}$$
  Through (18) and (19), a dependence results between the amplitude $\sqrt{\rho }$ and phase s of the state function Ψ in the form:
  (31) $$\rho =\rho_{0}e^{-2s}$$
  with $\rho_{0}$ an integration constant.
(c) The vectorial field B is uniform, a situation in which the potential vector A has the expression:
  (32) $$A=\frac{1}{2}\left(r\times B\right)$$

In particular, for one-dimensional dynamics of any complex system, $A_{x}$ can be chosen in the form:

(33) $$A_{x}=\frac{1}{2}Bx,B=\mathrm{const}.$$

Since, on the basis of the above hypotheses (a)–(c), Equation (29) takes the form:

(34) $$J_{x}=-\lambda {\left(dt\right)}^{\left[\frac{2}{f\left(\alpha \right)}\right]-1}\frac{d\rho }{dx}-\frac{1}{2}gBx\rho$$

Equation (27) becomes the multifractal equation Fokker–Planck:

(35) $$\partial_{t}\rho +\partial_{x}\left(-\frac{1}{2}gBx\rho \right)+\partial_{xx}\left[-\lambda {\left(dt\right)}^{\left[\frac{2}{f\left(\alpha \right)}\right]-1}\rho \right]=0$$

Therefore, the correlations between the two descriptions, i.e., the multifractal Schrödinger and the multifractal Mandelung one, imply multifractal Fokker–Planck description of complex system dynamics.

With the notations:

(36) $$\eta =\frac{1}{2}gB,D=2\lambda {\left(dt\right)}^{\left[\frac{2}{f\left(\alpha \right)}\right]-1}$$

where η can be correlated with the strength of the hydrogel network and D is the fractalization degree, as previously defined, the solution of Equation (35) has the expression:

(37) $$\rho \left(x,t\right)={\left(\frac{1}{2\pi \left(D/\eta \right)\left[1-\exp(-2\eta t)\right]}\right)}^{1/2}\exp\left[-\frac{{\left[x-x_{0}\exp(-\eta t)\right]}^{2}}{2\left(D/\eta \right)\left[1-\exp(-2\eta t)\right]}\right]$$

signifying that the density of states is a multifractal Gaussian whose average multifractal value decreases exponentially to zero and whose multifractal variance tends asymptotically towards (D/η).

### 4.3. Implications of the Compatibility between the Two Models for Describing the Release Dynamics through Scale Relativity Theory. Model Validation

In the framework of the presented theoretical model, considering our aim to simplify the analysis of the release process, we will incorporate all the process variables, i.e., the synthesis parameters, that influence the hydrogel structure into one named fractalization degree.

The theoretical model established, through Equation (37), a direct correlation between the fractalization degree and the maximum efficiency of the polymer–drug system. Therefore, when calibrating the theoretical model on the experimental data of the release efficiency, the fractalization degree for each sample can be extracted (Table 5).

Regarding the issues mentioned previously related to unexpected behavior in respect to the dependence of released drug efficiency on the crosslinker amount, the values summarized in Table 6 show that the fractalization degree, which cumulates all the reaction parameters, can explain these, i.e., a proportionality between the fractalization degree and release efficiency.

**Figure 5:**
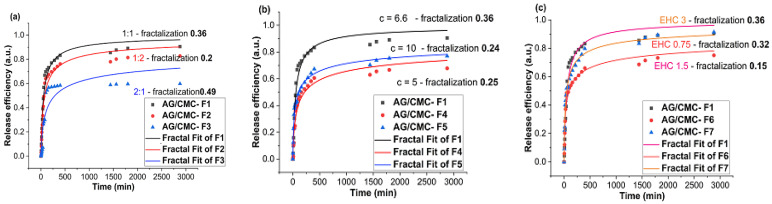
Comparative plots of fractal fits for different release kinetics: (**a**) F1, F2, F3 films, (**b**) F1, F4, F5 films, (**c**) F1, F6, F7 films.

This multivariable empirical approach emphasizes that, in the multifractal analysis plane, the fractalization degree of the system is one parameter that can quantify the overall outcome of all system interactions, generating a single parameter that can predict the behavior of certain polymer–drug combinations (Figure 5).

**Figure 6:**
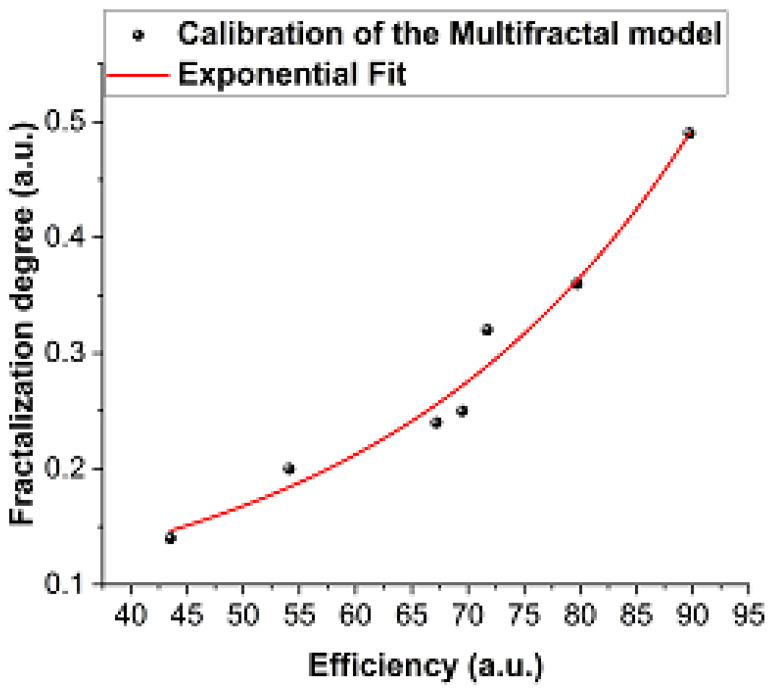
The calibration to the multifractal model.

The calibration of the model is described in Figure 6 where the efficiency of the system is plotted as a function of the fractalization degree as given by the model. An exponential function proved to be the best calibration fit.

We can conclude that the presented multifractal model is able to describe the system behavior in regard to the release efficiency through a global parameter, the fractalization degree, that encompasses all reaction parameters.

## 5. Conclusions

This study proposed the preparation of a new hydrogel film based on crosslinking with epichlorohydrin of alginates and carboxymethyl cellulose. The obtained films were characterized in terms of their swelling capacity and loading/release drug ability as well as in the way in which they were influenced by the synthesis reaction parameters, such as the polymer concentration and crosslinker amount. The films’ recovered fractions were high and ranged between 43% and 89%. The results showed that all films presented good swelling properties in water and a good ability to encapsulate and release the drug.

The drug release profiles revealed a fast release over 9 h followed by a slower phase (characterized by a constant release) until 48 h, with all values being in good concordance with the variable parameters considered. Therefore, we consider that these new hydrogel films have the potential to become systems for the controlled release of bioactive compounds with modulable properties in terms of released amount.

The proposed theoretical model analyzed the release process at the microscopic level and we described this process in regard to the release efficiency through a global parameter—the fractalization degree—associated with the system complexity. This encompasses all reaction parameters that influence the release process, thus, simplifying the release analysis. The calibration of the model on the experimental data revealed an exponential dependence of the release efficiency on the fractalization degree.

## Figures and Tables

**Table 1:** The initial reactants composition for hydrogel preparation and yields.

Sample Code	Concentration (%)	Polymer Ratio (g/g)	AG(g)	CMC(g)	NaOH (mL)	ECH (mL)	Yields (%)
F1	6.6	1:1	0.5	0.5	15	1.5	79.7
F2	15	1:2	0.5	1	15	1.5	54.1
F3	15	2:1	1	0.5	15	1.5	89.7
F4	10	1:1	0.5	0.5	10	1.5	69.5
F5	5	1:1	0.5	0.5	20	1.5	67.2
F6	6.6	1:1	0.5	0.5	15	0.75	71.7
F7	6.6	1:1	0.5	0.5	15	3	43.5

**Table 2:** The maximum swelling degree of the synthesized films.

	F1	F2	F3	F4	F5	F6	F7
Q, %	587	316	398	299	953	1273	362

**Table 3:** MT loaded and released film profiles.

	F1	F2	F3	F4	F5	F6	F7
MT-loaded films, mg	16.6	14.9	11.9	13.4	15.8	18.0	10.8
MT-loaded films Efficiency, %	44.63	50.22	60.39	55.40	47.48	39.86	63.99
MT-released films, mg/g	15.03	12.36	7.11	9.07	12.13	13.54	9.88
MT-released films Efficiency MT, %	90.49	82.78	59.83	67.82	76.97	75.04	91.41

**Table 4:** The dependence of the swelling, loading and releasing capacity on the crosslinker amount (the other reaction parameters are considered constant).

Sample Code	ECH Crosslinker (mL)	Swelling Ratio (%)	Loaded MT		Released MT	
			mg	%	mg	%
F6	0.75	1273	18.0	39.86	13.54	75.04
F1	1.5	587	16.6	44.63	15.03	90.49
F7	3	362	10.8	63.99	9.88	91.41

**Table 5:** Fractalization degree vs. reaction parameters.

Sample Code	Process Variables						Fractalization Degree
	Concentration (%)	Molar Ratio	AG (g)	CMC (g)	NaOH (mL)	ECH (mL)	
F1	6.6	1:1	0.5	0.5	15	1.5	0.36
F2	15	1:2	0.5	1	15	1.5	0.20
F3	15	2:1	1	0.5	15	1.5	0.49
F4	10	1:1	0.5	0.5	10	1.5	0.24
F5	5	1:1	0.5	0.5	20	1.5	0.25
F6	6.6	1:1	0.5	0.5	15	0.75	0.15
F7	6.6	1:1	0.5	0.5	15	3	0.32

**Table 6:** Fractalization degree vs. crosslinker amount and released drug amount.

Sample Code	ECH Crosslinker (mL)	Swelling Ratio (%)	Release MT (%)	Fractalization Degree
F6	0.75	1273	75.04	0.15
F1	1.5	587	90.49	0.36
F7	3	362	91.41	0.32

## Data Availability

The data presented in this study are available on request from the corresponding author.

## Supplementary Materials

The following are available online at https://www.mdpi.com/article/10.3390/polym13244461/s1, Figure S1: SEM images of the AG/CMC films of all samples F1–F7.
