# Supplementary figures and images for: Hydrogels Based on Alginates and Carboxymethyl Cellulose with Modulated Drug Release—An Experimental and Theoretical Study
Source: Polymers (Basel). 2021 Dec 20;13(24):4461. doi: 10.3390/polym13244461 (PMC8703298; doi:10.3390/polym13244461)

F1

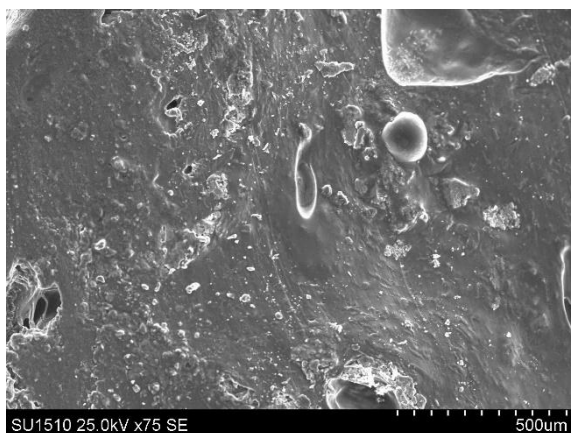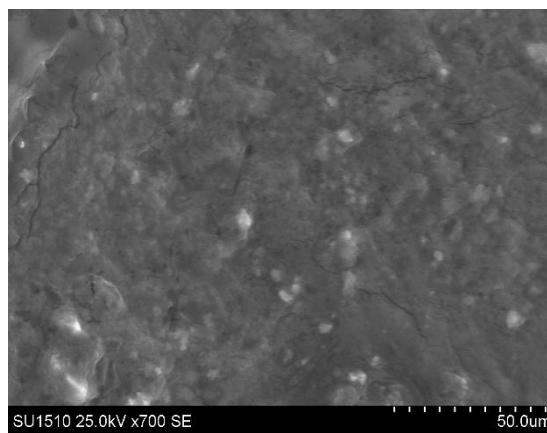

F2

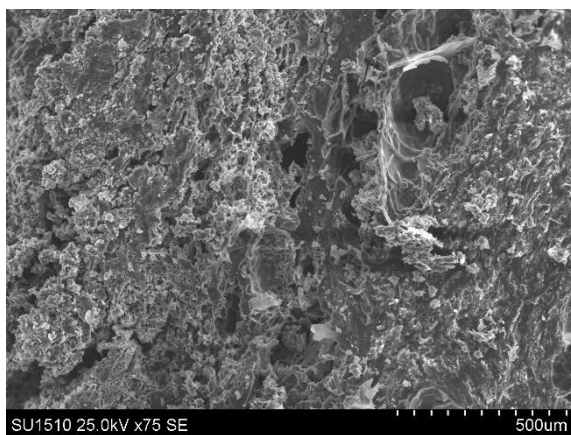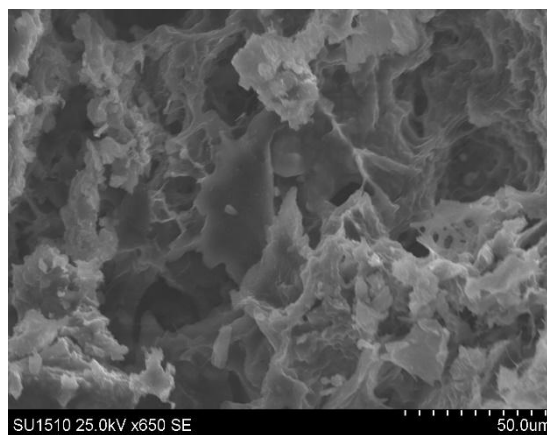

F3

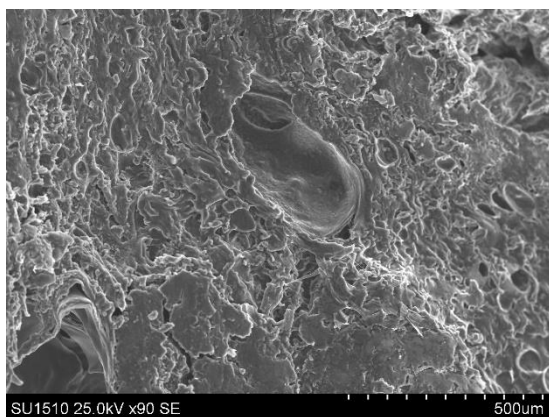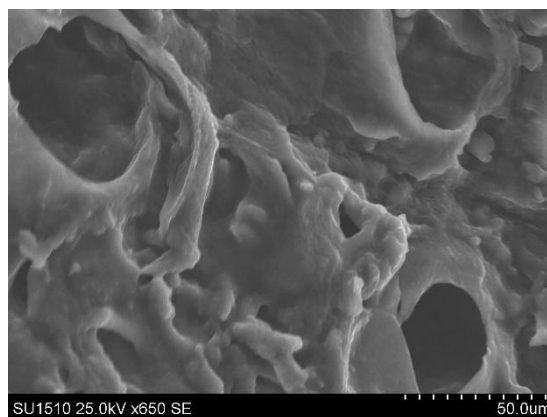

F4

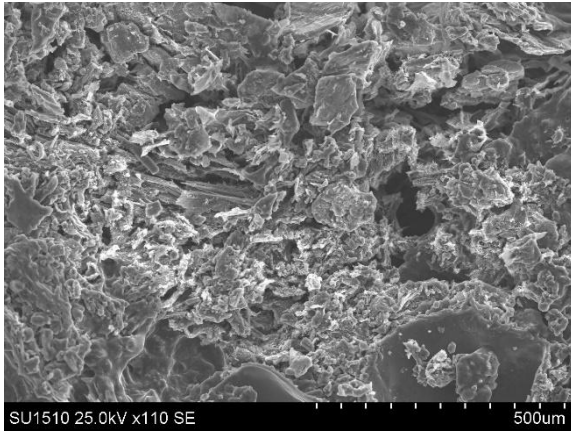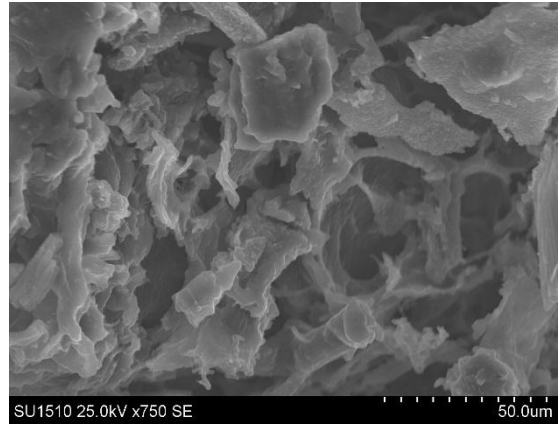

F5

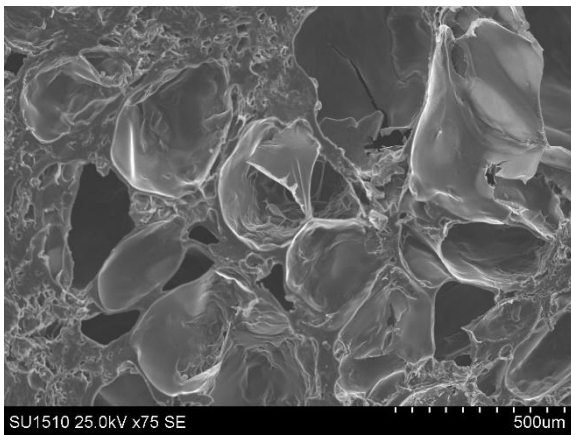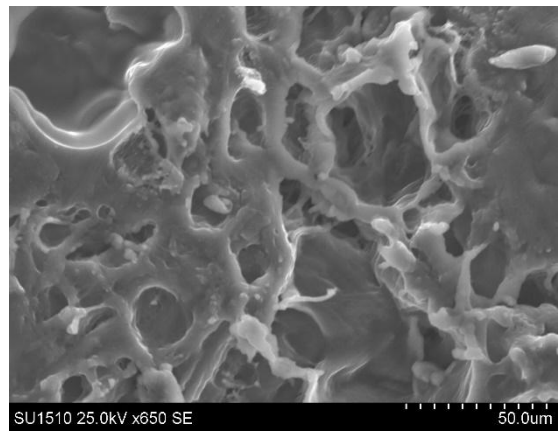

F6

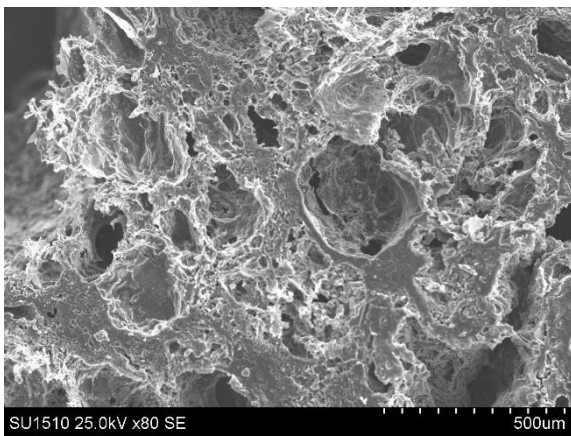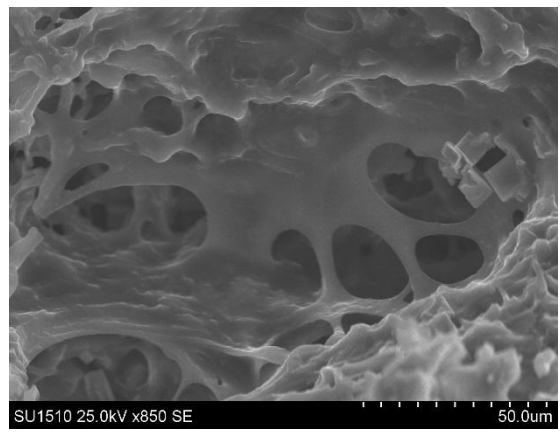

F7

Supplement: Supplementary file 1 [file polymers-13-04461-s001.zip › Figure S1.pdf]
